# Supplementary material for: OpenMM 7: Rapid development of high performance algorithms for molecular dynamics
Source: PLoS Comput Biol. 2017 Jul 26;13(7):e1005659. doi: 10.1371/journal.pcbi.1005659 (PMC5549999; doi:10.1371/journal.pcbi.1005659)
Supplement: S1 Source Code — (ZIP) [file pcbi.1005659.s002.zip › OpenMM-7.0.1-Source/docs/api-python/generated/simtk.openmm.openmm.AmoebaAngleForce.html]

AmoebaAngleForce — OpenMM 7.0.1.dev-5e86c4f documentation


# AmoebaAngleForce¶

*class* `simtk.openmm.openmm.``AmoebaAngleForce`(*\*args*)¶
:   This class implements an interaction between triplets of particles that varies with the angle between them. The interaction is defined by a 6th order polynomial. Only the quadratic term is set per-angle. The coefficients of the higher order terms each have a single value that is set globally.

    To use it, create an AmoebaAngleForce object then call addAngle() once for each angle. After an angle has been added, you can modify its force field parameters by calling setAngleParameters(). This will have no effect on Contexts that already exist unless you call updateParametersInContext().

    `__init__`(*self*) → AmoebaAngleForce¶
    :   \_\_init\_\_(self, other) -> AmoebaAngleForce

        Create an AmoebaAngleForce.

    Methods

    |  |  |
    | --- | --- |
    | `__init__`((self) -> AmoebaAngleForce) | \_\_init\_\_(self, other) -> AmoebaAngleForce |
    | `addAngle`((self, particle1, particle2, ...) | Add an angle term to the force field. |
    | `getAmoebaGlobalAngleCubic`((self) -> double) | Get the global cubic term |
    | `getAmoebaGlobalAnglePentic`((self) -> double) | Get the global pentic term |
    | `getAmoebaGlobalAngleQuartic`((self) -> double) | Get the global quartic term |
    | `getAmoebaGlobalAngleSextic`((self) -> double) | Get the global sextic term |
    | `getAngleParameters`(self, index) | Get the force field parameters for an angle term. |
    | `getForceGroup`((self) -> int) | Get the force group this Force belongs to. |
    | `getNumAngles`((self) -> int) | Get the number of angle stretch terms in the potential function |
    | `setAmoebaGlobalAngleCubic`(self, cubicK) | Set the global cubic term |
    | `setAmoebaGlobalAnglePentic`(self, penticK) | Set the global pentic term |
    | `setAmoebaGlobalAngleQuartic`(self, quarticK) | Set the global quartic term |
    | `setAmoebaGlobalAngleSextic`(self, sexticK) | Set the global sextic term |
    | `setAngleParameters`(self, index, particle1, ...) | Set the force field parameters for an angle term. |
    | `setForceGroup`(self, group) | Set the force group this Force belongs to. |
    | `updateParametersInContext`(self, context) | Update the per-angle parameters in a Context to match those stored in this Force object. |
    | `usesPeriodicBoundaryConditions`((self) -> bool) | Returns whether or not this force makes use of periodic boundary conditions. |

    `getNumAngles`(*self*) → int¶
    :   Get the number of angle stretch terms in the potential function

    `setAmoebaGlobalAngleCubic`(*self*, *cubicK*)¶
    :   Set the global cubic term

        |  |  |
        | --- | --- |
        | Parameters: | **cubicK** (*double*) – the cubic force constant for the angle |

    `getAmoebaGlobalAngleCubic`(*self*) → double¶
    :   Get the global cubic term

        |  |  |
        | --- | --- |
        | Returns: | global cubicK term |
        | Return type: | double |

    `setAmoebaGlobalAngleQuartic`(*self*, *quarticK*)¶
    :   Set the global quartic term

        |  |  |
        | --- | --- |
        | Parameters: | **quarticK** (*double*) – the quartic force constant for the angle |

    `getAmoebaGlobalAngleQuartic`(*self*) → double¶
    :   Get the global quartic term

        |  |  |
        | --- | --- |
        | Returns: | global quartic term |
        | Return type: | double |

    `setAmoebaGlobalAnglePentic`(*self*, *penticK*)¶
    :   Set the global pentic term

        |  |  |
        | --- | --- |
        | Parameters: | **penticK** (*double*) – the pentic force constant for the angle |

    `getAmoebaGlobalAnglePentic`(*self*) → double¶
    :   Get the global pentic term

        |  |  |
        | --- | --- |
        | Returns: | global penticK term |
        | Return type: | double |

    `setAmoebaGlobalAngleSextic`(*self*, *sexticK*)¶
    :   Set the global sextic term

        |  |  |
        | --- | --- |
        | Parameters: | **sexticK** (*double*) – the sextic force constant for the angle |

    `getAmoebaGlobalAngleSextic`(*self*) → double¶
    :   Get the global sextic term

        |  |  |
        | --- | --- |
        | Returns: | global sextic term |
        | Return type: | double |

    `addAngle`(*self*, *particle1*, *particle2*, *particle3*, *length*, *quadraticK*) → int¶
    :   Add an angle term to the force field.

        |  |  |
        | --- | --- |
        | Parameters: | - **particle1** (*int*) – the index of the first particle connected by the angle - **particle2** (*int*) – the index of the second particle connected by the angle - **particle3** (*int*) – the index of the third particle connected by the angle - **length** (*double*) – the equilibrium angle, measured in degrees - **quadraticK** (*double*) – the quadratic force constant for the angle, measured in kJ/mol/radian^2 |
        | Returns: | the index of the angle that was added |
        | Return type: | int |

    `getAngleParameters`(*self*, *index*)¶
    :   Get the force field parameters for an angle term.

        |  |  |
        | --- | --- |
        | Parameters: | **index** (*int*) – the index of the angle for which to get parameters |
        | Returns: | - **particle1** (*int*) –   the index of the first particle connected by the angle - **particle2** (*int*) –   the index of the second particle connected by the angle - **particle3** (*int*) –   the index of the third particle connected by the angle - **length** (*double*) –   the equilibrium angle, measured in degrees - **quadraticK** (*double*) –   the quadratic force constant for the angle, measured in kJ/mol/radian^2 |

    `setAngleParameters`(*self*, *index*, *particle1*, *particle2*, *particle3*, *length*, *quadraticK*)¶
    :   Set the force field parameters for an angle term.

        |  |  |
        | --- | --- |
        | Parameters: | - **index** (*int*) – the index of the angle for which to set parameters - **particle1** (*int*) – the index of the first particle connected by the angle - **particle2** (*int*) – the index of the second particle connected by the angle - **particle3** (*int*) – the index of the third particle connected by the angle - **length** (*double*) – the equilibrium angle, measured in degrees - **quadraticK** (*double*) – the quadratic force constant for the angle, measured in kJ/mol/radian^2 |

    `updateParametersInContext`(*self*, *context*)¶
    :   Update the per-angle parameters in a Context to match those stored in this Force object. This method provides an efficient method to update certain parameters in an existing Context without needing to reinitialize it. Simply call setAngleParameters() to modify this object’s parameters, then call updateParametersInContext() to copy them over to the Context.

        The only information this method updates is the values of per-angle parameters. The set of particles involved in an angle cannot be changed, nor can new angles be added.

    `usesPeriodicBoundaryConditions`(*self*) → bool¶
    :   Returns whether or not this force makes use of periodic boundary conditions.

        |  |  |
        | --- | --- |
        | Returns: | true if nonbondedMethod uses PBC and false otherwise |
        | Return type: | bool |

    `__delattr__`¶
    :   x.\_\_delattr\_\_(‘name’) <==> del x.name

    `__format__`()¶
    :   default object formatter

    `__getattribute__`¶
    :   x.\_\_getattribute\_\_(‘name’) <==> x.name

    `__hash__`¶

    `__reduce__`()¶
    :   helper for pickle

    `__reduce_ex__`()¶
    :   helper for pickle

    `__sizeof__`() → int¶
    :   size of object in memory, in bytes

    `__str__`¶

    `getForceGroup`(*self*) → int¶
    :   Get the force group this Force belongs to.

    `setForceGroup`(*self*, *group*)¶
    :   Set the force group this Force belongs to.

        |  |  |
        | --- | --- |
        | Parameters: | **group** (*int*) – the group index. Legal values are between 0 and 31 (inclusive). |

High performance molecular simulation on GPUs


### Navigation

- Application Layer
- Library Layer
  - Core Objects
  - Forces
  - Integrators
  - Extras

©2015, Stanford University and the Authors.
|
Powered by Sphinx 1.3.1
& Alabaster 0.7.7
|
Page source
